# Feasibility of Using High-Contrast Grating as a Point-of-Care Sensor for Therapeutic Drug Monitoring of Immunosuppressants

**DOI:** 10.1109/JTEHM.2020.2966478

**Published:** 2020-01-30

**Authors:** Yi-Cheng Liu, Christina Thantrakul, Shu Kan, Connie Chang-Hasnain, Dong-Ru Ho

**Affiliations:** 1 Department of Electrical Engineering and Computer SciencesUniversity of California–Berkeley 1438 BerkeleyCA94720USA; 2 Department of BioengineeringUniversity of California–Berkeley 1438 BerkeleyCA94720USA; 3 Center for Cardiovascular TechnologyDepartment of Cardiovascular MedicineStanford University 6429 StanfordCA94305USA; 4 Division of UrologyDepartment of SurgeryChang Gung Memorial HospitalChiayi61363Taiwan; 5 Graduate Institute of Clinical Medical Sciences, Chang Gung University 56081 Taoyuan City33302Taiwan; 6 Department of NursingChang Gung University of Science and Technology 63113 Chiayi61363Taiwan

**Keywords:** Point-of-care, metamaterials, transplantation, therapeutic drug monitoring

## Abstract

Point-of-care (POC) testing has demonstrated great transformative potential in personalized medicine. In particular, patients undergoing transplantation require POC testing to ensure appropriate serum immunosuppressant levels so as to maintain adequate graft function and survival. However, no suitable POC device for monitoring immunosuppressant levels is currently available. Exploiting the latest advances in metamaterials can lead to a breakthrough in POC testing. A high-contrast grating (HCG) biosensor is a low-cost, compact, simple-to-fabricate, and easy-to-operate structure. It is highly sensitive and robust in surface-based biomarker detection, which is favorable for the efficiency of a POC device. In this study, the feasibility of using an HCG as a POC sensor for therapeutic drug monitoring of immunosuppressants was evaluated. The detection efficiency of the most commonly prescribed immunosuppressive medication cyclosporine A by using this sensor was demonstrated to be comparable to those of conventional commercial kits, suggesting that the sensor has the potential to be used as a rapid detection and feedback platform for increasing drug compliance and improving new organ transplant survival.

## Graphical abstract

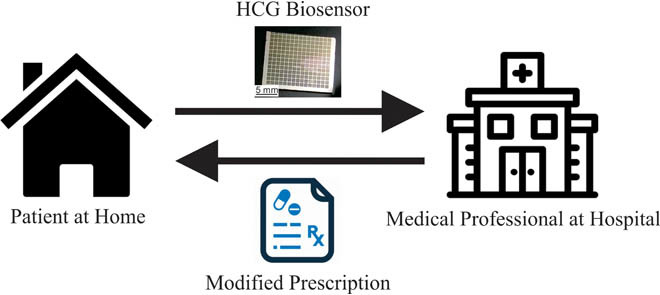

## I. Introduction

Transplantation is the ultimate treatment for organ failure [1]–[5]. According to the Global Observatory on Donation and Transplantation, in 2015, approximately 126,670 patients underwent organ transplantation worldwide, and this number is increasing at a rate of 5.8%. On average, 22 people die every day while waiting for a transplant. In 2014, 4,761 patients died while waiting for a kidney transplant and 3,668 patients became too sick to receive a kidney transplant. In Taiwan, 8,948 patients are currently waiting for organ transplantation, of which nearly 7000 patients await kidney transplants. In the United States, currently, 121,678 patients are waiting for lifesaving organ transplants, of which 100,791 patients await kidney transplants. In 2016, 178 kidney transplantations were conducted in Taiwan, and in 2014, 17,107 kidney transplants were conducted in the United States. Organ shortage and the consequent high cost are becoming serious concerns. Saving grafts benefits not only patients but also doctors and insurance companies, thereby saving a considerable percentage of the health care budget. Moreover, maintaining adequate graft function and survival is crucial.

With the introduction of immunosuppressants, the success rate of organ transplantation has drastically increased. According to the Taiwan National Health Insurance Database, the cumulative number of patients who received kidney transplantation with maintenance immunosuppressant therapy is approximately 8000 [6]. To ensure adequate graft function and graft survival, patients must maintain appropriate serum immunosuppressant levels. The current renal transplantation therapy employs several immunosuppressive agents, mostly in combination with other agents, commonly classified according to their mechanism of action: Calcineurin inhibitors [cyclosporine A (CsA) and tacrolimus], inhibitors of purine synthesis (mycophenolate mofetil/mycophenolic acid and azathioprine), and mammalian target of rapamycin inhibitors (sirolimus and everolimus). These drugs are frequently co-administered with glucocorticoids (e.g., methylpredinosolone and prednisolone), and during induction therapy, with monoclonal or polyclonal antibodies (e.g., basiliximab and thymoglobulin) [7]. However, preventing immunosuppressant overdose is essential. Excess immunosuppression may result in morbidity and mortality, including infection, neoplasm, and toxicity [8], [9]. Immunosuppressants cause irreversible kidney damage at high doses [9]. In clinical practice, these drugs have a narrow therapeutic index and significant interindividual variability in the blood, which are affected by factors such as drug–nutrient interactions, drug–disease interactions, renal insufficiency, inflammation and infection, sex, age, polymorphism, and liver mass. According to a study, 48.1% of death-censored graft failures resulted from noncompliance [10].

**FIGURE 1.**
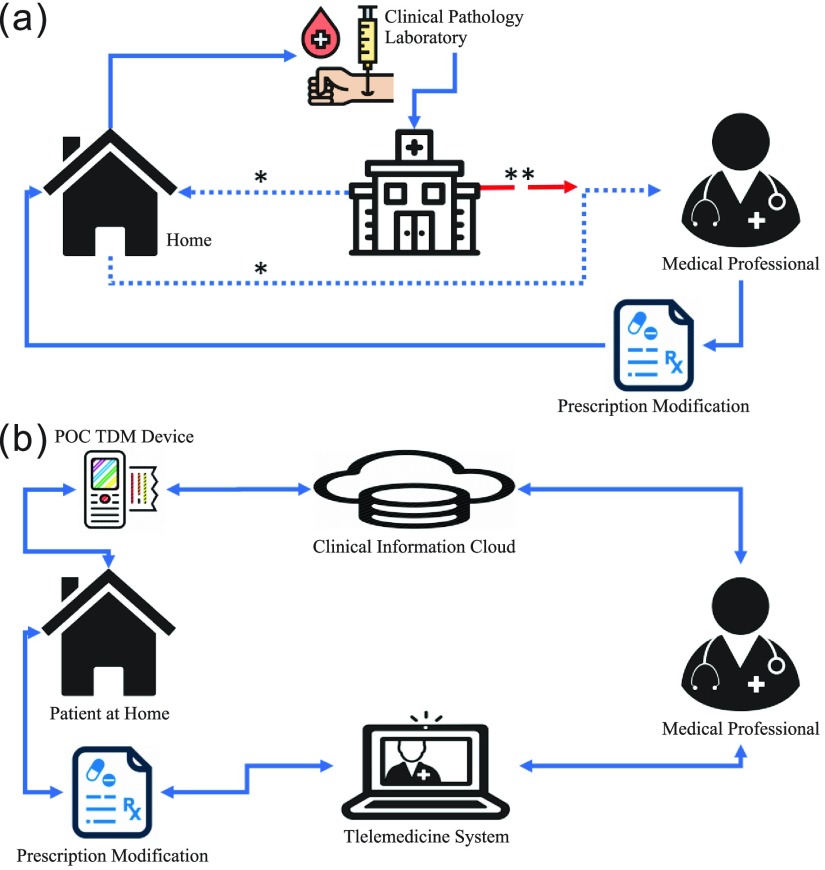
Comparisons between current and new clinical workflows. (a) Current clinical workflow: The patient visits a hospital to take blood tests and then waits for the results; this process may take several days. *: The dotted line indicates a patient’s commute from their home to the hospital and back; this process may take several days in low-volume facilities for waiting the clinical pathology results to be ready. **: The segmented line indicates the process in high-volume transplantation centers, wherein the laboratory results are obtained within 6 hours and patients can thus receive their modified prescriptions in the same visit. (b) New clinical workflow based on our concept: By using a POC TDM device, the patient measures the drug levels at home and subsequently uploads the data to the cloud. The appropriate medical professional reviews these data in their office and recommends any further action if required.

Practical direct evaluation of immune cell response or proliferation through immunosuppressant therapeutic drug monitoring (TDM) has been increasingly used. Immunosuppressant TDM is widely practiced, particularly for cyclosporine, tacrolimus, sirolimus, and mycophenolic acid [11]. Traditional methods of immunosuppressant measurement include enzyme-linked immunosorbent assay (ELISA), radioimmunoassay, high-performance liquid chromatography, and tandem mass spectrometry. In general, these methods require large, expensive laboratory equipment and highly trained personnel [12]. They consequently involve long-duration incubation and wash steps requiring fluidic control, which increases the complexity and cost of the device used. The current immunosuppressant TDM method requires a patient to visit the health care provider on a monthly basis because of the miscellaneous process illustrated in Fig. 1a. For a more cost-effective manner, the clinical pathology laboratory performs the analysis after a bunch of blood samples is collected, which may take several days. However, to achieve ideal immune function, TDM must be performed more frequently. Studies have proposed some methods for optimizing the immunosuppressant treatment, including the choice of sample matrix for monitoring [13], inconsistent assay performance [14], variable absorption of the drug from the original formulation [15], and poor correlation between trough concentration and clinical effects [16]. Moreover, over time, the absorption of immunosuppressants becomes erratic and incomplete, resulting in high intrapatient and interpatient variability. Therefore, POC becomes essential in optimizing the immunosuppressant performance [17], [18].

POC biomarker detection has been performed using optical microcavities [19], [20], photonic crystals [21], metal nanohole arrays [22], and surface plasmon resonance [23]. However, the devices required in this process are fabricated using an expensive e-beam lithography process and have limited utility in remote testing because of the need for high-precision optical alignments performed by trained technicians. A high-contrast grating (HCG) is a label-free optical biosensor with a spectral linewidth of approximately 500 pm and is sensitive to ligand-induced changes in surface properties [24]. The small footprint and high efficiency of an HCG structure can be suitable for wavelength conversion in chip-scale integrated optics. Laser light can be directly coupled to an HCG resonator from a fiber output with a coupling efficiency of as high as 87%, which provides a simple and robust alignment system [25]. Moreover, the resonance quality factor and refractive index sensitivity of an HCG structure are higher than those of other metamaterials that also use direct surface-normal coupling [20]–[22], [26]. This structures has been proposed and demonstrated in the detection of biomarkers and biomaterials [24], [27], [28]. To prove its practicality in clinical settings, we evaluated the feasibility of an HCG as a POC sensor for immunosuppressant TDM. In vitro experiments were conducted, and the detection of the most common immunosuppressant CsA was demonstrated.

## II. Materials and Methods

### A. High-Contrast Grating Biosensor

**FIGURE 2.**
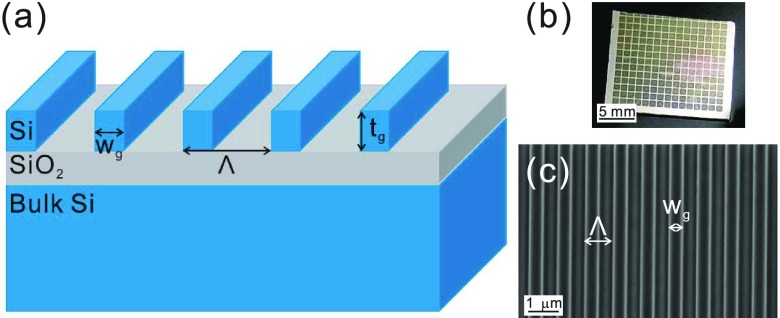
(a) Illustration of an HCG; the period ($\Lambda $), duty cycle ($\eta =\,\,\text{W}_{g}/\Lambda $), and thickness ($\text{t}_{g}$) determine its properties. (b) Photograph of the HCG array. Each mesh represents an HCG device. (c) Scanning electron microscope image of gratings within a single fabricated HCG.

A one-dimensional high-contrast metastructure composed of a material (silicon) with a high optical index of refraction was surrounded by a material with a low optical index of refraction such as air. The periodic form of this construction is called an HCG and is presented in Fig. 2a. The HCG may be designed to be a broadband reflector, a broadband transmitter, or a high-quality factor resonator by changing the period ($\Lambda $), duty cycle ($\eta =\,\,\text{W}_{g}/\Lambda $), thickness ($\text{t}_{g}$), and materials used, where the resonance is established inside the grating layer with the air/Si and Si/oxide interfaces acting as cavity boundaries. By using a grating layer of appropriate thickness, the two resonance modes can be allowed to interfere constructively at the entrance and exit boundaries. This feature yields a high-Q resonator, with the light propagating in a direction that is normal to the grating plane. A detailed discussion of the physics and design of one-dimensional high-contrast metastructures has been presented in previous publications [25], [29]–[31].

The devices were patterned onto a 6-in silicon-on-insulator wafer with an Si-layer thickness of 500 nm (from SOITEC). The devices were fabricated using DUV (ASML 300) lithography, followed by silicon refractive ion etching (Lam Research), which are both standard processes in semiconductor manufacturing. Fig. 2b shows the photograph of the fabricated HCG chip, wherein each mesh represents an HCG device. The scanning electron microscopy image shown in Fig. 2c evidences a clear grating structure. The HCG biosensor, precisely fabricated using silicon optical lithography and etching, can generate high-efficiency, specific and strong resonances that are sensitive to changes in surface properties and can be monitored using surface-normal excitation. Its sensitivity depends on the quality factor (Q) of the resonator, which could be designed to be as high as 14000 [29]. However, the higher the Q is, the narrower is the resonant linewidth; this necessitate a more precise alignment. Consequently, we selected an appropriate design to perform our experiment. Accordingly, the HCG devices used in this study had the following parameters: $\eta =0.55$, $\Lambda =784$ nm; $\eta =0.56$, $\Lambda =776$ nm; $\eta =0.54$, $\Lambda =808$ nm; $\eta =0.54$, $\Lambda =792$ nm; $\eta =0.52$, $\Lambda =824$ nm; $\eta =0.53$, $\Lambda =816$ nm; $\eta =0.49$, $\Lambda =864$ nm; $\eta =0.45$, $\Lambda =912$ nm; and $\eta =0.45$, $\Lambda =904$ nm, while $\text{t}_{g}$ was fixed to 500 nm. Although each device was slightly different, which resulted in different resonant center wavelengths, they all had a similar quality factor.

A compact and dense self-assembled monolayer of silicon nitride (Si_3_N_4_) with a thickness of 234 Å was deposited on the surface of the devices through plasma-enhanced chemical vapor deposition. For a waveguide, an insulator with a refractive index sufficiently higher than the surrounding medium is essential to confine evanescent waves so as to enhance the detection sensitivity. Therefore, Si_3_N_4_ was used due to its high refractive index, good compromise with low leakage current, and low conductivity [32]. Salinization of the Si_3_N_4_ layer was proposed [33]–[35], which facilitates the immobilization of antibodies. The details are described in Section II.B. The sensitivity was reported as 418 nm/RIU in our previous study [24].

### B. Antibody Immobilization

The surface of the HCG is processed through antibody immobilization to form an antibody–antigen binding assay. The binding of the antigen to the HCG surface results in a red-shift of the resonance wavelength, which is a consequence of the effective increase in the length of the optical cavity. The monoclonal antibody is covalently linked to the HCG surface by using maleimide–thiol coupling. In this study, 1 mg/mL of antibody solution was combined with $100~\mu \text{M}$ maleimidobenzoic acid succinimide ester (MBS) in phosphate buffered saline (PBS) for 2 h. Thereafter, a PD-10 column was used to remove unlabeled MBS. Simultaneously, the HCG resonator was submerged in a 5% solution of (3-mercaptopropyl) trimethoxysilane in IPA for 1 h. This thiol salinized the sensor surface and facilitated the formation of stable thiol-ether bonds between the sensor surface and the MBS antibody. The sensor was rinsed with PBS and then submerged in the 1 mg/mL MBS–antibody solution for 2 h. After another rinse with PBS, the sensor was immersed in PBS containing 3% bovine serum albumin to reduce nonspecific binding of proteins to the sensor surface. The sensor was washed again, and its reflection spectrum was recorded using the measurement setup described in the following section.

### C. Reflection Spectrum Measurements for Quantitative Analysis

**FIGURE 3.**
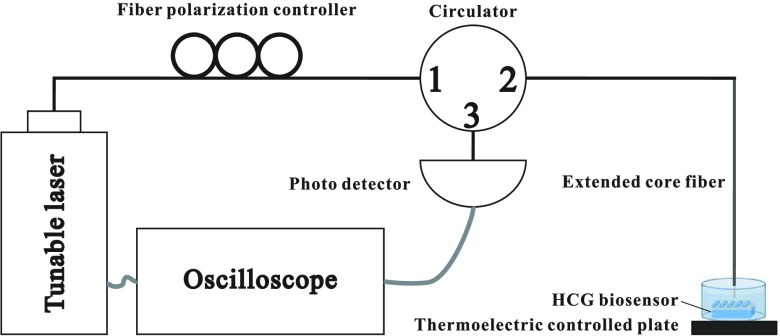
Experimental setup. Light travels from a tunable laser through the fiber polarization controller and is directed to the HCG biosensor by the circulator. The light reflected from the HCG biosensor is directed to the photo detector by the circulator. An oscilloscope synchronized with the tunable laser and the photo detector is used to record the signal.

The HCG biosensor was placed inside a small watertight container fixed on a plate set thermoelectrically controlled to 20°C. The sample solution in the container was probed with surface normal incident light from a tunable laser (Agilent Tunable Laser Module 81680A) by using an extended-core, single-mode optical fiber placed close to the sensor surface. The light from the laser traversed a single-mode optical fiber to a fiber polarization controller (Thorlabs) and was then incident on the biosensor. The biosensor was placed on a multidimensional stage to ensure good coupling. The fiber tip was immersed inside a target solution to avoid reflection at the solution surface. The light reflected by the sensor was collected by the same optical fiber, and a circulator was used to direct the reflected light to a detector. The reflected optical power of the sensor over the range of wavelengths of interest was recorded using an indium–gallium–arsenide (InGaAs) photodetector (Thorlabs DET10) synchronized to the tunable laser with an oscilloscope. The setup is illustrated in Fig. 3. The sensor was then immersed in PBS containing the antigen of interest in the relevant quantity for 10 min. The sensor was washed once more with the solvent, and its reflection spectrum was recorded.

To characterize the performance of the HCG sensor in detecting target antigens, we tested a complementary antibody–antigen pair composed of unlabeled IgG from rabbit serum (Sigma-Aldrich, I5006) as the antigen and a polyclonal antibody directed against rabbit IgG that was produced in goat (Sigma-Aldrich, R2004) as the detection antibody. Solutions ($200~\mu \text{L}$) of the rabbit IgG in PBS were added separately and sequentially to the HCG surface over a concentration ranging from $10^{-5}$ to $10{^{3}}~\mu \text{g}$/mL. The reflection spectrum was recorded after the addition of each antigen sample. The HCG surface immobilized with CsA monoclonal antibody (Santa Cruz Biotechnology, sc-80997) and its response to the CsA (Santa Cruz Biotechnology, sc-3503) were demonstrated using the same procedure. Because CsA is insoluble in water, it was dissolved in DMSO and then diluted with the appropriate matrix, either PBS or serum.

## III. Results and Discussion

**FIGURE 4.**
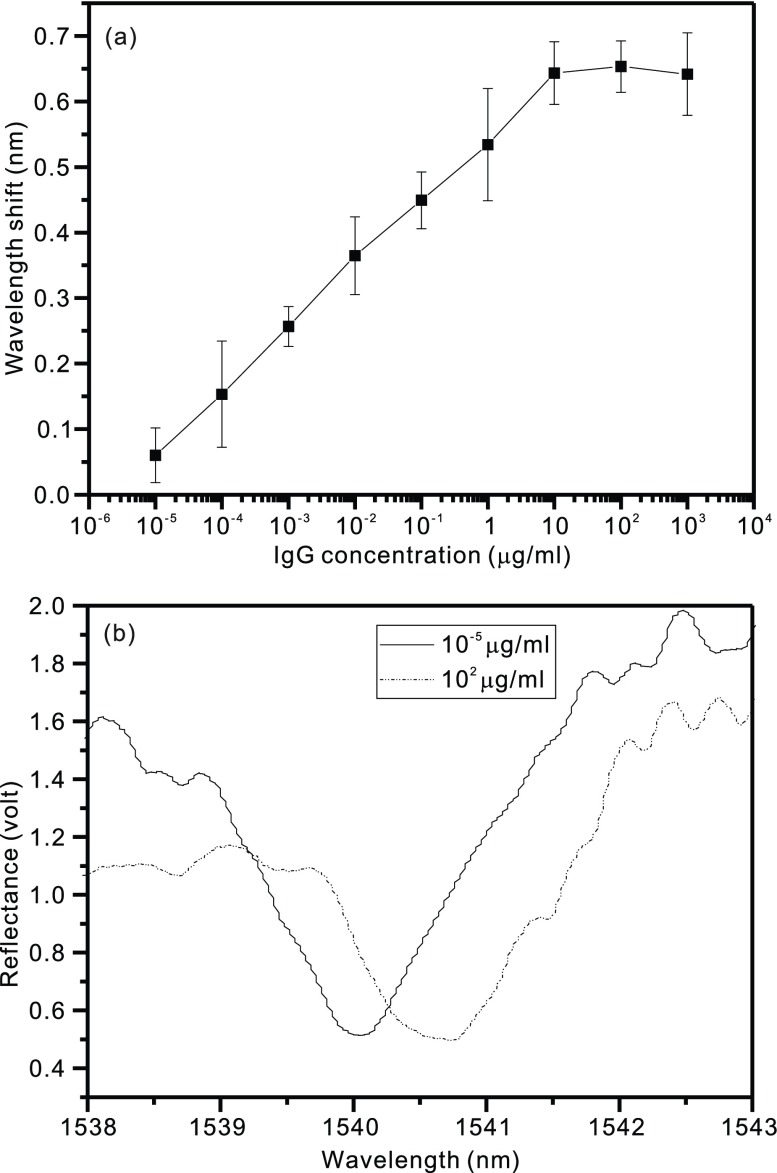
Resonant wavelength shifts (a) and spectra (b) for different concentrations of purified rabbit IgG added to an HCG resonator surface coated with antirabbit IgG. Each point was obtained by averaging three measurements.

Fig. 4 presents the results of the reaction between the rabbit IgG antibody and rabbit IgG. Fig. 4a shows that the red-shift in the resonance wavelength increased with an increase in the concentration of rabbit IgG. Each point is obtained by averaging three measurements. The resonant wavelength shift is related to the increased optical length caused by the antibody–antigen complex on the HCG surface. This optical length changes the boundary condition of the optical cavity between grating bars and results in a red-shift in the resonant wavelength [24]. The signal associated with rabbit IgG binding to the surface-coupled rabbit IgG antibody saturated at approximately $10~\mu \text{g}$/mL. The lowest detectable concentration of rabbit IgG recorded for this device was $10^{-4}\,\,\mu \text{g}$/mL, which resulted in a red-shift in the resonance wavelength of 93 pm. Fig. 4b shows the spectrum of the wavelength deep shifting as the rabbit IgG concentration increases. This result is in agreement with our previous work [24], and it can serve as the positive control to confirm the results of the immunosuppressant experiment. The quality factor (Q) was estimated as 1800 by Q $=\,\,\text{f}_{r}/\Delta \text{f}$, where $\text{f}_{r}$ is the resonant frequency and $\Delta \text{f}$ is the full width at half maximum.

**FIGURE 5.**
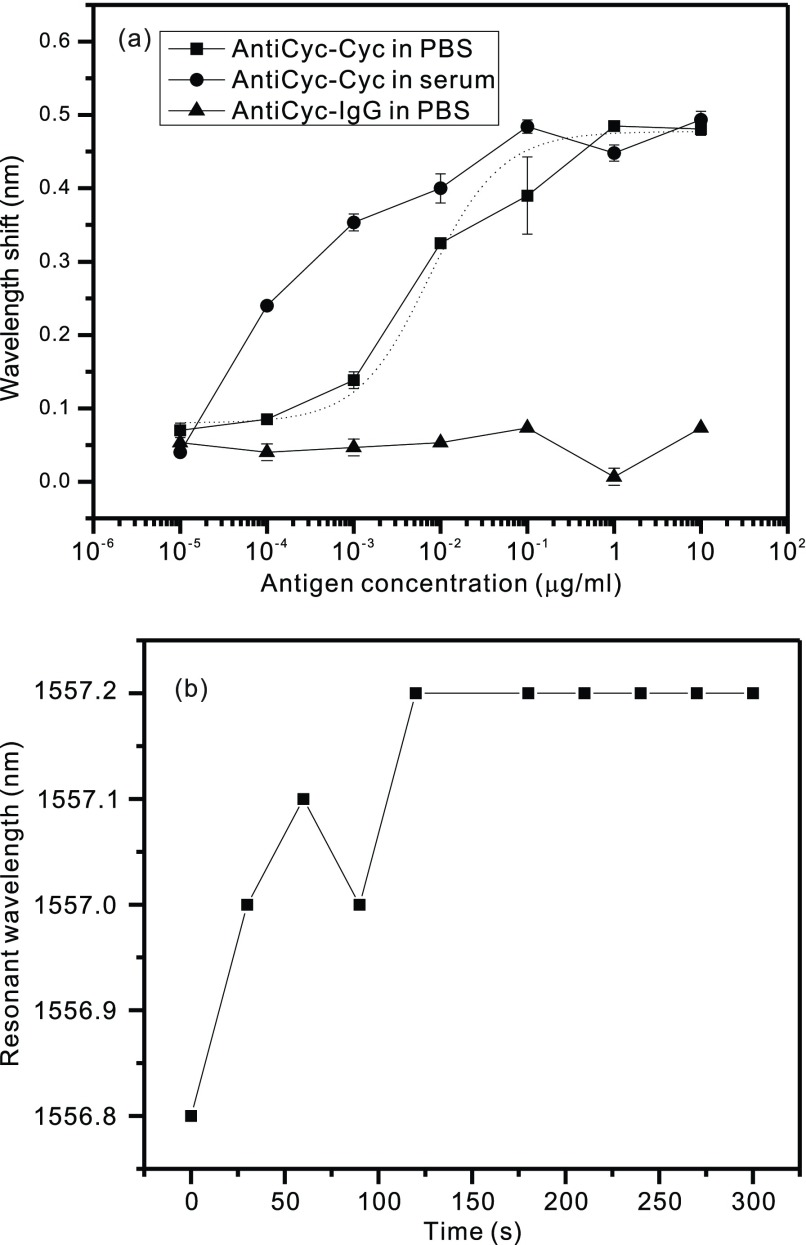
(a) Measurement results for the CsA molecule inside PBS (square), serum (circle), and negative control (triangle). Each point was obtained by averaging three measurements. (b) Real-time recording of the resonant wavelength of purified CsA ($0.1~\mu \text{g}$/mL) in PBS added to an HCG resonator surface coated with anti-CsA.

For CsA measurement, the result of the resonant wavelength shift as a function of the concentrations is shown in Fig. 5a. A distinct redshift in the resonant wavelength of the sensor was observed with an increase in the CsA concentration, indicating binding between the CsA antibody and CsA. The same experiment was performed in the serum, as shown in Fig. 5a, and a similar redshift trend was observed. In clinical practice, the detection limit for CsA is 20 ng/mL [36]. The results reveal that serum CsA levels of 0.1 ng/mL can be detected, which is adequately sensitive for the detection of blood CsA levels associated with immunosuppressant TDM. The solubility of CsA in DMSO is $10^{-1}\,\,\mu \text{g}$/mL, with a larger variation (the square dot). Subsequently, the curve leveled off. To determine whether nonspecific binding influences the sensor’s response, the measurement was performed using the antibody specific to CsA and the rabbit IgG antigen. The results illustrated in Fig. 5a indicate no significant resonant wavelength shift, which provides convincing evidence that the binding between the CsA antigen and CsA antibody causes the resonant wavelength shift. To determine the time taken for completion of the binding reaction, we recorded the resonant wavelength every 30 s for 5 min after the addition of $0.1~\mu \text{g}$/mL of CsA in PBS. Fig. 5b indicates the resonant wavelength as a function of time. The signal associated with antigen binding increases and reaches a plateau within 150 s. Similar results were observed in tacrolimus measurements (data not shown).

A comparison of Figs. 4a and 5a revealed that the IgG measurement exhibited a linear curve. Because the molecular weight of IgG (150 kDa) is considerably larger than that of CsA (1.2 kDa), the evanescent wave penetration depth is a value between the sizes of the two analytes. This result also reflects the different amplitudes of the error bars. In the detection of serum CsA, the higher shift results from the residue attached on the HCG surface. Because of the weak antibody affinity, the sensor cannot be washed many times. The antibody affinity can be further improved specifically to achieve immobilization, targeting and coupling the targeted analyte by using various methods [37]. The time to complete the wavelength shift is dominated by the time to achieve thermal equilibrium. Because the resonant wavelength is sensitive to temperature variations, we installed a thermoelectric controller to achieve a steady state in less than 3 min. Nevertheless, the resonant wavelength can be designed to be highly sensitive to the thickness of the antibody–antigen complex but insensitive to temperature variations [28]. The control experiments verified the specificity/selectivity of the HCG biosensor, which is a critical requirement in the development of clinically relevant assays of disease biomarkers. The limit of detection (LOD) was estimated to be 0.54 ng/mL by using the formula, LOD = 3N/S, where N is the standard deviation of the noise and S is the sensitivity. This LOD is superior to those of several newly developed immunoassays addressed in a recent review [36].

In addition to the high quality factor of HCG, the employed tunable laser has a high wavelength accuracy of ±0.01 nm. Therefore, such an expensive instrument may not favor the POC requirement. The HCG biosensor is fabricated using a standard silicon manufacturing process that allows for the fabrication of more than 10,000 unique sensors on a single square-centimeter of chip that can be further embellished with integrated microfluidic and electronic systems. The integration of a stand-alone tunable laser and photodiode helps reduce the cost of the measurement system, and the surface-normal fiber detection format overcomes the requirement in other competing devices for continuous adjustments to maintain a precise optical alignment. A suitable design addressed in a recent report [38] placed the edge of the resonance spectrum alongside the edge of the excitation spectrum. This makes the resonance spectrum to shift, resulting in an intensity change of the reflection light. In this case, the system uses only the photodetector instead of a bulky and expensive tunable laser. In addition, graphene can be incorporated in the system for ultra-speed operation [39] and a GaN-based membrane with the resonant wavelength in the visible region can be added [40].

Because of the trend of polypharmacy in perioperative stages of organ transplantation, obtaining drug level profiles is essential. Polypharmacy includes a combination of different calcineurin inhibitors, mammalian target of rapamycin inhibitors, proliferation inhibitors (inhibitors of inosine monophosphate dehydrogenase), and steroids. Integrating a fiber array, an extension of the parallel screening of multiple biomarkers, provides the possibility of obtaining all drug levels using a single test [24]. Thus, we conclude that the HCG biosensor has the potential to become a practical POC device for immunosuppressant TDM.

## IV. Conclusion

This study bridges the knowledge gap between technology and clinical practice. We demonstrated the quantitative detection of CsA and achieved detection limits comparable to those reported using commercial immunosuppressant TDM kits. Close monitoring of critical biomarkers, such as blood glucose monitoring in diabetes patients and immunosuppressant monitoring in transplantation patients, influences their quality of life and incidence of complications. The techniques reported herein could be readily used in the development of POC devices for other immunosuppressants, such as tacrolimus, mycophenolic acid, azathioprine, sirolimus, and everolimus. The application of such POC devices can reduce costs and rapidly provide clinicians with information regarding drug efficacy in individual patients. Furthermore, the use of an automated clinical management system can help reduce the frequency of rejection and toxicity episodes [41]. In future studies, the HCG sensor could be developed as a rapid detection and feedback platform; a related concept is illustrated in Fig. 1b. Subjecting a patient to frequent telemonitoring can increase their drug compliance and new organ survival and thus improve their quality of life.

## Disclosures

The authors have no financial interests regarding the manuscript and do not have any other potential conflicts of interest to disclose.
